# Bone marrow lesions predict site-specific cartilage defect development and volume loss: a prospective study in older adults

**DOI:** 10.1186/ar3209

**Published:** 2010-12-29

**Authors:** Dawn Dore, Ashleigh Martens, Stephen Quinn, Changhai Ding, Tania Winzenberg, Guangju Zhai, Jean-Pierre Pelletier, Johanne Martel-Pelletier, François Abram, Flavia Cicuttini, Graeme Jones

**Affiliations:** 1 Menzies Research Institute Tasmania, University of Tasmania, Private Bag 23, Hobart, 7000, Australia; 2 Department of Epidemiology and Preventive Medicine, Monash University, 89 Commercial Road, Melbourne, 3004, Australia; 3 Department of Twin Research and Genetic Epidemiology, King's College London, St Thomas' Hospital, Westminster Bridge Road, London, SE1 7EH, UK; 4 Osteoarthritis Research Unit, University of Montreal Hospital Research Centre (CRCHUM), Notre-Dame Hospital, 1560 Sherbrooke St. East, Montreal, QC H2L 4M1, Canada; 5 Arthro Vision Inc., 1560 Rue Sherbrooke East, Montreal, Quebec H2K 1B6, Canada

## Abstract

**Introduction:**

Recent evidence suggests that bone marrow lesions (BMLs) play a pivotal role in knee osteoarthritis (OA). The aims of this study were to determine: 1) whether baseline BML presence and/or severity predict site-specific cartilage defect progression and cartilage volume loss; and 2) whether baseline cartilage defects predict site-specific BML progression.

**Methods:**

A total of 405 subjects (mean age 63 years, range 52 to 79) were measured at baseline and approximately 2.7 years later. Magnetic resonance imaging (MRI) of the right knee was performed to measure knee cartilage volume, cartilage defects (0 to 4), and BMLs (0 to 3) at the medial tibial (MT), medial femoral (MF), lateral tibial (LT), and lateral femoral (LF) sites. Logistic regression and generalized estimating equations were used to examine the relationship between BMLs and cartilage defects and cartilage volume loss.

**Results:**

At all four sites, baseline BML presence predicted defect progression (odds ratio (OR) 2.4 to 6.4, all *P *< 0.05), and cartilage volume loss (-0.9 to -2.9% difference per annum, all *P *< 0.05) at the same site. In multivariable analysis, there was a significant relationship between BML severity and defect progression at all four sites (OR 1.8 to 3.2, all *P *< 0.05) and BML severity and cartilage volume loss at the MF, LT, and LF sites (β -22.1 to -42.0, all *P *< 0.05). Additionally, baseline defect severity predicted BML progression at the MT and LF sites (OR 3.3 to 3.7, all *P *< 0.01). Lastly, there was a greater increase in cartilage volume loss at the MT and LT sites when both larger defects and BMLs were present at baseline (all *P *< 0.05).

**Conclusions:**

Baseline BMLs predicted site-specific defect progression and cartilage volume loss in a dose-response manner suggesting BMLs may have a local effect on cartilage homeostasis. Baseline defects predicted site-specific BML progression, which may represent increased bone loading adjacent to defects. These results suggest BMLs and defects are interconnected and play key roles in knee cartilage volume loss; thus, both should be considered targets for intervention.

## Introduction

Bone marrow lesions (BMLs), detected by magnetic resonance imaging (MRI), have been recognized as an important feature in knee osteoarthritis (OA) [1,2]. A number of studies have linked BMLs with knee pain [1,3-5] although other studies have failed to demonstrate such a relationship [6-8]. Baseline BMLs and increases in BML size have been shown to predict cartilage defect progression [9-12] and cartilage loss [9,10,13-18]. However, most of these studies have used a compartment-level approach by combining tibial and femoral sites [9,10,13-15] and/or medial and lateral tibiofemoral compartments [9,10]. The relationship between BMLs and changes in site-specific cartilage has only recently been examined [16-18]. Kothari *et al. *found that the presence of BMLs at baseline was associated with cartilage loss in the same subregion at two years [18]. In another study, Roemer *et al. *examined BML changes with changes in cartilage over time [17]. They reported that the absence of BMLs at baseline and follow-up was associated with a decreased risk of adjacent cartilage loss, while new or progressive BMLs displayed a high risk of adjacent cartilage loss [17]. Cartilage scores in both of these studies were assessed using the Whole-Organ Magnetic Resonance Imaging Score (WORMS) method, which semi-quantitatively scores cartilage integrity by using one scale for both cartilage defects and cartilage loss. Alternatively, Raynauld *et al. *examined the subregional relationship between BMLs with a quantitative measure of cartilage volume loss and found that an increase in bone oedema was associated with cartilage volume loss in the same subregions of the medial but not in the lateral compartment [16]. Therefore, there is increasing evidence to demonstrate that BMLs predict site-specific cartilage changes; however, it remains unclear whether BMLs at one site predict cartilage changes in another.

There is an ongoing debate about the role BMLs play in the development of cartilage damage and loss. It remains unclear whether BMLs precede, accompany, or follow cartilage damage and volume loss in OA [18]. Many studies have shown that baseline BMLs predict subsequent cartilage damage and/or loss [9-11,13-15,18]; however, to the best of our knowledge, there have been no studies examining whether baseline cartilage defects predict BML progression.

Therefore, the aims of this population-based longitudinal study were to examine: 1) the relationship between baseline BMLs and site-specific changes in cartilage (defects and/or volume changes); 2) whether baseline BMLs at one site predict cartilage changes (defects and/or volume changes) in another; and 3) whether baseline cartilage defects predict site-specific BML progression.

## Materials and methods

### Subjects

This study was conducted as part of the Tasmanian Older Adult Cohort (TASOAC) study, an ongoing prospective, population-based study that was initiated in 2002 and was aimed at identifying the environmental, genetic, and biochemical factors associated with the development and progression of OA at multiple sites (hand, knee, hip, and spine). Subjects between the ages of 50 and 80 years were randomly selected from the electoral roll in Southern Tasmania (population 229,000), with an equal number of men and women. The overall response rate was 57%. Subjects who were institutionalized were excluded from the study. All research conducted within this manuscript is in compliance with the Helsinki Declaration and was approved by the Southern Tasmanian Health and Medical Human Research Ethics Committee. All subjects gave informed written consent.

The current study consists of a sample of 405 participants who had MRI measures at baseline and follow-up. The range of follow-up was 2.0 to 4.7 years (mean: approximately 2.7 years). The majority of participants (90%) were followed up between 2.2 to 3.2 years.

### Anthropometrics

Weight was measured to the nearest 0.1 kg (with shoes, socks, and bulky clothing removed) using a single pair of electronic scales (Seca Delta Model 707, Bradford, MA, USA). Height was measured to the nearest 0.1 cm (with shoes and socks removed) using a stadiometer. Body mass index (BMI) was calculated (kg/m^2^).

### Magnetic Resonance Imaging

An MRI of the right knee was acquired with a 1.5T whole-body magnetic resonance unit (Picker, Cleveland, OH, USA) using a commercial transmit-receive extremity coil. Image sequence included the following: (1) a T1-weighted fat saturation three-dimensional (3-D) gradient recall acquisition in the steady state, flip angle 30°, repetition time 31 ms, echo time 6.71 ms, field of view 16 cm, 60 partitions, 512 × 512-pixel matrix, acquisition time 5 minutes 58 seconds, one acquisition; sagittal images were obtained at a partition thickness of 1.5 mm without between-slice gap; (2) a T2-weighted fat saturation 3-D fast spin echo, flip angle 90°, repetition time 3,067 ms, echo time 112 ms, field of view 16 cm, 15 partitions, 228 × 256-pixel matrix; sagittal images were obtained at a partition thickness of 4 mm with a between-slices gap of 0.5 to 1.0 mm.

### Cartilage morphology evaluation

Knee tibial cartilage volume was assessed by a trained observer on T1-weighted MR images at baseline and follow-up by means of image processing on an independent workstation using Osiris software (University of Geneva, Geneva, Switzerland) as previously described [19,20]. The volumes of individual cartilage plates (medial tibia and lateral tibia) were isolated from the total volume by manually drawing disarticulation contours around the cartilage boundaries on a section by section basis. These data were then re-sampled by means of bilinear and cubic interpolation (area of 312 × 312 mm and 1.5 mm thickness, continuous sections) for the final 3-D rendering. The coefficient of variation (CV) was 2.1% for the medial tibia and 2.2% for the lateral tibia [19]. Knee femoral cartilage volume was determined by means of image processing on an independent workstation using Cartiscope™ (ArthroVision Inc., Montreal, QC, Canada), as previously described [21-23]. The segmentation of the cartilage-synovial interfaces was carried out with the semi-automatic method under reader supervision and with corrections when needed. Cartilage volume was evaluated directly from a standardized view of 3D cartilage geometry as the sum of elementary volumes. The CV was approximately 2% [21]. The cartilage volume assessment was done for the medial and lateral condyles delineated by the Blumensaat's line [22]. Absolute change in cartilage volume was calculated as: follow-up cartilage volume - baseline cartilage volume. Rate of change in cartilage volume was calculated as: percentage change per annum (pa) = 100*((absolute change/baseline cartilage volume)/time between two scans in years).

Cartilage defects were assessed by a trained observer at baseline and follow-up on T1-weighted MR images (score range, 0 to 4) at the tibial and femoral sites, medially and laterally, as previously described [24] as follows: grade 0 = normal cartilage; grade 1 = focal blistering and intracartilaginous low-signal intensity area with an intact surface and base; grade 2 = irregularities on the surface or base and loss of thickness <50%; grade 3 = deep ulceration with loss of thickness >50%; and grade 4 = full-thickness chondral wear with exposure of subchondral bone. A cartilage defect also had to be present on at least two consecutive slices. The cartilage was considered to be normal if the band of intermediate signal intensity had a uniform thickness. If more than one defect was present on the same site the highest score was used. Intraobserver repeatability was assessed in 50 subjects with at least one week between the two measurements with intraclass correlation coefficients (ICC) of 0.93, 0.92, 0.95, and 0.80 at the medial tibia, medial femur, lateral tibia, and lateral femur, respectively. Cartilage defect progression was defined as an increase of one or more on the 0- to 4-point scale. Those whose scores remained the same or decreased by one or more were defined as stable or decreasing.

### Subchondral BML evaluation

Subchondral BMLs were assessed by a trained observer at baseline and followed-up on T2-weighted MR images and defined as areas of increased signal adjacent to the subcortical bone at the medial tibial, medial femoral, lateral tibial, and lateral femoral sites. Each BML was scored on the basis of lesion size (for example, a lesion was scored as grade 1 if it was only present on one slice, grade 2 if present on two consecutive slices, or grade 3 if present on three or more consecutive slices). The BML with the highest score was used if more than one lesion was present at the same site. Intraobserver repeatability was assessed in 50 subjects with at least a one-week interval between the two readings with ICCs of 0.94, 1.00, 0.89 and 0.96 at the medial tibia, medial femur, lateral tibia, and lateral femur, respectively. BML progression was defined as an increase of one or more on the 0- to 3-point scale. Those whose scores remained the same or decreased by one or more were defined as stable or decreasing.

In an extended observation, BMLs were also scored using a modified version of WORMS by a separate research group, in order to compare the two scoring systems. Briefly, BMLs were assessed on T1-weighted MR images and the joint was divided into its anatomical regions (medial and lateral condyle, medial and lateral tibial plateau, and patella), which were further subdivided into anterior, central, and posterior for the femur, and medial and lateral for the patella and the tibial plateaus. Subchondral bone marrow abnormalities were then assessed comparing the surface of the lesion with the surface of the subregion in the corresponding image. If the lesion was depicted in multiple slides, the one with the largest extent was chosen. When the lesion is oriented along the latero-medial direction, a reconstructed axial image is used for the evaluation. A scale from 0 to 3 was used, where 0 = absence, 1 = < 25%, 2 = 25% to 50%, and 3 = > 50% of this ratio. The central and posterior femoral subregions and the tibial plateau formed the medial and lateral compartments. The medial and lateral anterior femoral subregions and the two patellar subregions formed the femoropatellar compartment. The inter-reader reliability of this BML scoring system has previously been shown to be excellent [16].

### Meniscal damage evaluation

Meniscal damage evaluation at baseline was performed by a trained observer as previously described [23]. In brief, the proportion of the menisci affected by the tear or extrusion was separately scored on the medial and lateral edges of the tibiofemoral joint space using a semi-quantitative scale. For tears the following scale applied: 0 = no damage, 1 = one of three areas involved (anterior, middle, posterior horns), 2 = two of three involved, 3 = all three areas involved. The extent of meniscal extrusion, not including the osteophytes, was evaluated for the anterior, middle, and posterior horns of the menisci in which 0 = no extrusion, 1 = partial extrusion and 2 = complete extrusion with no contact with the joint space (severe).

Cartilage volume measurements, cartilage defects, BMLs, and meniscal damage scoring were all done independently of one another.

### Statistical analysis

Site-specific associations were defined as the associations within the same site (example, the association between medial tibial BMLs and medial tibial defect increases). Compartment-specific associations were defined as the associations within the same compartment (for example, the association between medial tibial BMLs and medial femoral defect increases).

T-tests and chi-square tests were used to compare differences in means and proportions where appropriate. Due to a lack of variation in baseline cartilage defect score in this cohort, cartilage defects were dichotomized for some analyses. Defect scores of 0 to 1 were coded 0 and of 2 to 4 were coded 1.

Logistic regression modeling was used to examine the site and compartment-specific associations between baseline BMLs with increases in cartilage defects (increase versus no increase) and baseline defects with increases in BMLs (increase versus no increase), after adjustment for age, sex, BMI, and defects if BMLs and BMLs if defects. As there is increasing evidence to suggest that meniscal damage plays an important role in disease progression, models were further adjusted for meniscal damage. Meniscal damage has been shown to predict cartilage loss [15,23] and BML development [25,26]. Therefore, it is believed that meniscal pathology, cartilage damage, and BMLs are all related, although the time sequence of these pathological events is still unclear. By further adjusting for meniscal damage we were able to assess whether the associations between BMLs and cartilage defects were independent of meniscal pathology. Due to the uncertainty of the chronological order of these features, we have chosen to display both the unadjusted and adjusted results. Standard diagnostic checks of model adequacy and unusual observations were performed. Hosmer-Lemeshow tests were performed to assess goodness-of-fit.

Generalized estimating equations (GEE) were used to examine the site and compartment-specific associations between baseline BMLs and cartilage defects with change in absolute cartilage volume after adjustment for age, sex, BMI, baseline site-specific cartilage volume, and defects if BMLs and BMLs if defects. Models were then further adjusted for meniscal damage to assess the independent effects of BMLs and cartilage defects on cartilage volume loss. The interaction between baseline BMLs and baseline defects on cartilage volume loss was also examined.

A *P-*value less than 0.05 (two-tailed) was considered statistically significant. All statistical analyses were performed on Intercooled Stata 10.0 for windows (StataCorp, College Station, TX, USA).

## Results

### Subjects

A total of 1,100 subjects (51% female) aged between 51 and 81 (mean: 63 years) participated in the TASOAC study. The current study consists of a sample of 405 participants who had MRI measures at baseline and follow-up. MRI scans were discontinued after this sample due to decommissioning of the MRI scanner. There were no significant baseline differences in demographics, cartilage defects, BMLs, and cartilage volume between the rest of the cohort and the subjects included in the current study.

The characteristics of the study sample by presence or absence of baseline BMLs at any site are presented in Table 1. At all four sites, in unadjusted analysis, subjects who had a BML at baseline had a higher prevalence of baseline cartilage defects, lost more cartilage volume from baseline to follow-up, and a higher proportion of them increased in cartilage defects from baseline to follow-up, compared with those subjects who did not have a BML at baseline. There was limited variation in baseline cartilage defect scores. No participants scored zero at the medial or lateral tibial sites. The majority of participants scored 1 and smaller numbers of participants scored ≥2 at all four sites.

**Table 1:** Characteristics of participants according to presence or absence of BMLs at baseline at each site*

	**Medial tibial**		**Medial femoral**		**Lateral tibial**		**Lateral femoral**	
				
	**BML absent**	**BML present**	**BML absent**	**BML present**	**BML absent**	**BML present**	**BML absent**	**BML present**
	**(*n *= 352)**	**(*n *= 53)**	**(*n *= 358)**	**(*n *= 47)**	**(*n *= 379)**	**(*n *= 26)**	**(*n *= 356)**	**(*n *= 49)**
	
Age (year)	63.2 (7.2)	63.5 (7.2)	63.3 (7.3)	62.7 (6.3)	63.1 (7.3)	65.0 (6.4)	63.3 (7.2)	63.0 (7.6)
Male sex (%)	49	53	49	57	50	46	**47**	**65†**
BMI (kg/m^2^)	27.6 (4.4)	28.0 (4.8)	27.5 (4.5)	28.5 (4.0)	27.7 (4.5)	26.6 (2.7)	27.7 (4.5)	27.2 (4.1)
Cartilage defects present baseline^# ^(%)	**7**	**28‡**	**15**	**43‡**	**15**	**38‡**	**5**	**31‡**
Cartilage defect increase (%)	**13**	**25†**	**23**	**44‡**	**14**	**52‡**	**17**	**41‡**
Cartilage volume baseline (mL)	2,332 (580)	2,352 (563)	3,949 (1,135)	4,024 (1,089)	2,763 (681)	2,687 (807)	4,327 (1,194)	4,351 (876)
Cartilage volume loss per annum (%)	**-2.3 (5.3)**	**-4.4 (5.1)†**	**-1.1 (2.1)**	**-2.2 (2.9)‡**	**-1.8 (4.0)**	**-4.7 (6.2) ‡**	**-0.8 (2.0)**	**-1.7 (1.9)†**
BML increase (%)	11	17	**5**	**15†**	11	12	**12**	**27‡**

*Mean (standard deviation) except for percentages. Bold denotes a statistically significant result. *P-*values determined by t-test or chi-square test (where appropriate).

† *P *< 0.05.

‡ *P *< 0.01.

#Defined as grade 2 or higher.

BMI, body mass index; BMLs, bone marrow lesions; mL, millilitre.

### BMLs and cartilage defects

#### Site-specific associations

**Figure 1:**
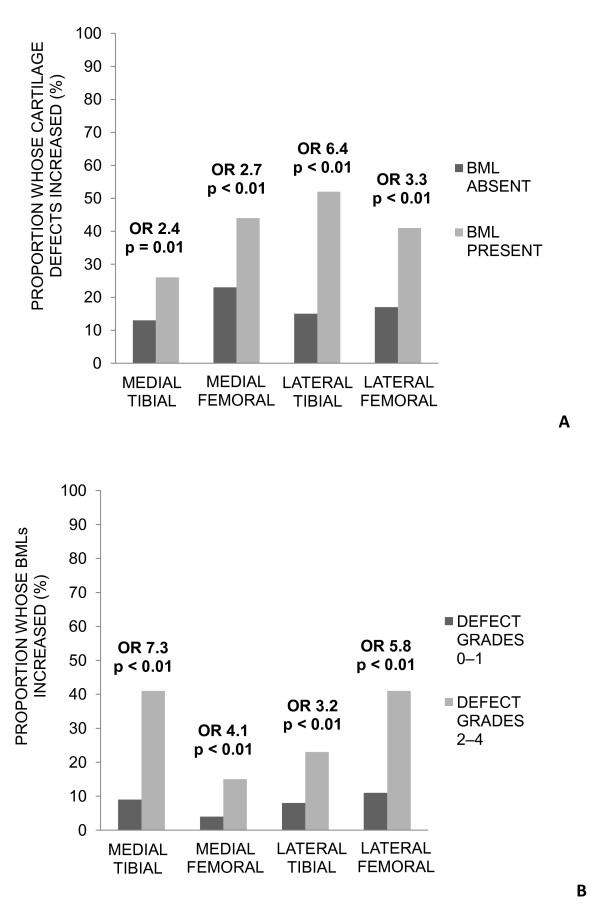
**Baseline BMLs with cartilage defect increases and baseline cartilage defects with BML increases by site**. **(a) **Proportion of participants whose cartilage defects increased in those with no baseline BML versus those with a baseline BML. **(b) **Proportion of participants whose BMLs increased in those with baseline cartilage defect grades 0 to 1 versus those with baseline cartilage defect grades 2 to 4.

Figure 1 describes the site-specific univariate relationship between (a) baseline BMLs and cartilage defect increases and (b) baseline cartilage defects and BML increases. There were a higher proportion of participants whose cartilage defects increased in those with a BML at baseline versus those without a BML at baseline (a). There were also a higher proportion of participants whose BMLs increased in those with baseline defect grades 2 to 4 versus those with defect grades 0 to 1 (b).

Table 2 describes the multivariable relationship between baseline BML severity and cartilage defect increases and baseline cartilage defect severity and BML increases. BMLs predicted site-specific cartilage defect increases in a dose-response fashion at each site, even after further adjustment for meniscal damage. For example, at the medial tibial site, the odds of a cartilage defect increasing opposed to not increasing was 1.8 times more per grade increase in baseline BML score. Cartilage defect severity predicted site-specific increases in BMLs in a dose-response manner also at each site; however, after further adjustment for meniscal damage this only persisted at the medial tibial and lateral femoral sites.

**Table 2:** Association between BMLs and cartilage defects

	Multivariable OR (95% CI)†	Multivariable OR (95% CI)‡
*BMLs predicting defect increases*		
Medial tibial BMLs	1.8 (1.2, 2.7)**	1.8 (1.1, 2.9)*
Medial femoral BMLs	2.3 (1.5, 3.5)**	2.2 (1.4, 3.5)**
Lateral tibial BMLs	2.8 (1.8, 4.5)**	3.2 (1.9, 5.4)**
Lateral femoral BMLs	3.3 (2.1, 5.0)**	3.0 (1.9, 4.8)**
*Defects predicting BML increases*		
Medial tibial defects	3.7 (2.1, 6.5)**	3.3 (1.6, 6.8)**
Medial femoral defects	2.2 (1.3, 3.8)**	2.0 (1.0, 4.1)
Lateral tibial defects	2.5 (1.5, 4.2)**	1.6 (0.8, 3.4)
Lateral femoral defects	2.6 (1.6, 4.2)**	3.7 (1.9, 7.3)**

Baseline BMLs (0 to 3) and site-specific increases in cartilage defects at the same site and baseline cartilage defects (0 to 4) and site-specific increases in BMLs at the same site.

Bold denotes a statistically significant result. *P < 0.05, **P < 0.01.

† Adjusted for age, sex, body mass index and baseline site-specific defects if BMLs and site-specific BMLs if defects. All *P*-values < 0.01.

‡ Further adjusted for meniscal extrusion and meniscal tear.

BMLs, bone marrow lesions; CI, confidence interval; OR, odds ratio.

#### Compartment-specific associations

Medial femoral BMLs predicted medial tibial cartilage defect increases (OR 1.7, 95% CI 1.1 to 2.7), and this persisted after further adjustment for medial tibial BMLs and meniscal damage (OR 1.9, 95% CI 1.2 to 3.0). BMLs did not significantly predict compartment-specific defect increases at any other site.

Lateral tibial defects predicted lateral femoral BML increases (OR 2.3, 95% CI 1.5 to 3.7), and this persisted after further adjustment for lateral femoral defects and meniscal damage (OR 2.3, 95% CI 1.1 to 4.7). Defects did not significantly predict compartment-specific BML increases at any other site.

### Cartilage volume loss

#### Site-specific associations

**Figure 2:**
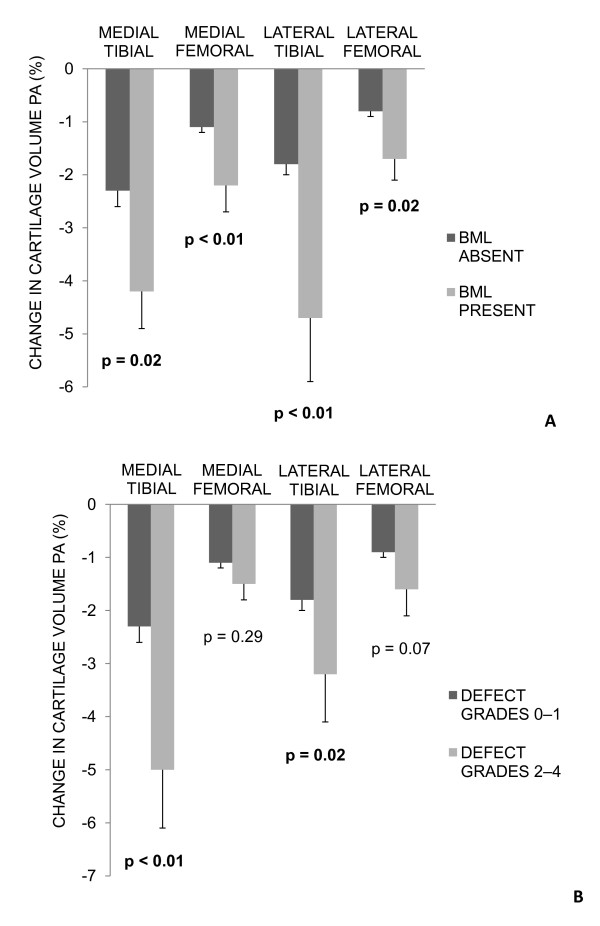
**Baseline BMLs and cartilage defects with cartilage volume loss (% per annum)**. **(a) **Mean cartilage volume loss of participants with no BML at baseline versus those with a BML at baseline. **(b) **Mean cartilage volume loss of participants with baseline cartilage defect grades 0 to 1 versus those with baseline defect grades 2 to 4. Error bars represent standard error.

Figure 2 describes the univariate relationship between (a) baseline BMLs and (b) baseline cartilage defects with cartilage volume loss at each site. Cartilage volume loss was higher in those participants with a baseline BML (a). Those participants with a baseline cartilage defect score ≥2 lost significantly more cartilage at the medial and lateral tibial sites (b).

Table 3 describes the multivariable relationship between baseline BML and cartilage defect severity with change in cartilage volume. BMLs predicted site-specific cartilage volume loss at all four sites in a dose-response fashion. After further adjustment for meniscal damage this persisted at the medial femoral, lateral tibial, and lateral femoral sites. Cartilage defects predicted cartilage volume loss at the medial tibial site only; however, this did not persist after adjustment for meniscal damage. At the medial femoral site cartilage defects trended towards predicting cartilage volume loss (*P *= 0.056).

**Table 3:** Baseline BMLs (0 to 3) and baseline cartilage defects (0 to 4) predicting absolute changes in cartilage volume

	Multivariable β (95% CI)†	Multivariable β (95% CI)‡
*Medial tibial*		
BMLs	-24.5 (-47.0, -2.0)*	-14.4 (-40.9, +12.1)
Cartilage defects	-33.7 (-60.3, -7.1)*	-5.0 (-43.6, +33.7)
*Medial femoral*		
BMLs	-42.0 (-63.6, -20.5)**	-42.0 (-63.7, -20.4)**
Cartilage defects	-17.2 (-34.7, +0.4)	-17.2 (-34.8, +0.4)#
*Lateral tibial*		
BMLs	-35.2 (-56.1, -14.4)**	-35.5 (-58.5, -12.6)**
Cartilage defects	-12.6 (-34.2, +9.0)	-21.7 (-50.2, +6.8)
*Lateral femoral*		
BMLs	-22.1 (-39.5, -4.7)*	-22.1 (-39.5, -4.7)*
Cartilage defects	-12.3 (-29.7, +5.1)	-12.3 (-29.7, +5.1)

Bold denotes a statistically significant result. *P < 0.05, **P < 0.01.

† Adjusted for age, sex, body mass index, baseline site-specific cartilage volume and defects if BMLs and BMLs if defects.

‡ Further adjusted for meniscal extrusion and meniscal tear.

#*P *= 0.056

β, beta-coefficient; BMLs, bone marrow lesions; CI, confidence interval.

**Figure 3:**
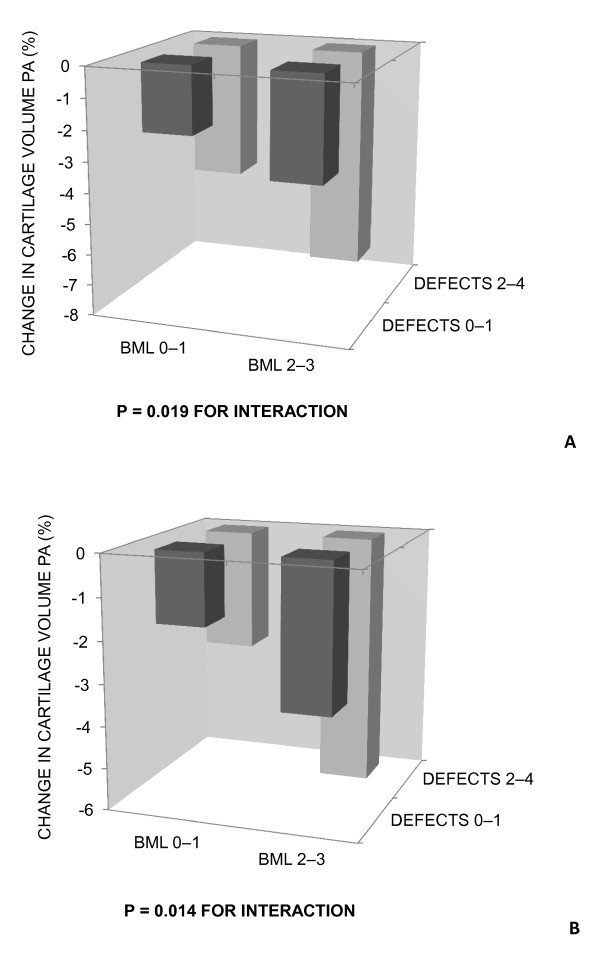
**Interaction between baseline BMLs and baseline cartilage defects on tibial cartilage volume loss (% per annum)**. There was a significant interaction between **(a) **medial tibial BMLs and medial tibial cartilage defects; and **(b) **lateral tibial BMLs and lateral tibial cartilage defects, for site-specific cartilage volume loss.

Figure 3 shows the interaction between baseline BMLs and cartilage defects on tibial cartilage volume loss. There was a higher rate of cartilage volume loss at both medial and lateral tibial sites when larger defects (grades 2 to 4) and BMLs (grades 2 to 3) were both present at the same site. There was no interaction between baseline BMLs and cartilage defects on femoral cartilage volume (data not shown).

#### Compartment-specific associations

Although BMLs predicted site-specific cartilage volume loss, they did not predict compartment-specific cartilage volume loss at any site (data not shown). For example, medial femoral BMLs did not predict medial tibial cartilage volume loss.

### Additional analysis

The results above were corroborated when BMLs were scored using the modified version of WORMS. Using the original scoring system BMLs predicted site-specific defect increases at all four sites (Table 2); whereas, using the WORMS system BMLs predicted site-specific defect increases at the medial femoral, lateral tibial, and lateral femoral sites (OR 2.9 to 13.7, all *P *< 0.05). Using the WORMS system BMLs predicted site-specific cartilage volume loss at the medial femoral, and lateral tibial sites (β -50.1 to -122.1, all *P *< 0.05); whereas, using the original scoring system BMLs also predicted cartilage volume loss at the lateral femoral site (Table 3).

## Discussion

This longitudinal study sheds light on the relationships between BMLs, cartilage defects, and cartilage volume loss. Baseline BMLs predicted site-specific cartilage defect progression and cartilage volume loss in a dose-response manner. To the best of our knowledge, this is the first study to show baseline cartilage defects predicted site-specific BML progression. Furthermore, there was an interaction between BMLs and cartilage defects on cartilage volume loss, with a much greater rate of tibial cartilage loss when both larger defects and BMLs were present at baseline.

Studies have only recently begun to examine the site-specific relationship between BMLs and cartilage changes [16-18]. We have demonstrated a site-specific relationship between BMLs and both cartilage defect progression and a quantitative measure of cartilage volume loss. We found that BMLs predicted cartilage defect progression and cartilage volume loss at all four sites (medial tibial, medial femoral, lateral tibial, and lateral femoral). After further adjustment for meniscal extrusions and tears, BMLs continued to predict cartilage defect progression at all four sites and cartilage volume loss at the medial femoral, lateral tibial, and lateral femoral sites, demonstrating the associations presented are independent of meniscal damage. Importantly our results demonstrate a dose-response relationship exists between BMLs and site-specific cartilage damage and volume loss. For every unit increase in BML size, the odds of a cartilage defect progressing increased and more cartilage volume was lost over time. This is very similar to a recent study by Tanamas *et al. *which showed that the severity of BMLs was positively associated with the risk of knee joint replacement in subjects with well established OA [27]. Although our study included those with and without OA, it suggests that the size of the BML is important at different stages. However, we are unaware of any study which shows that BML size increases with stage of OA.

This study is unique in that it also explored whether BMLs at one site predicted cartilage damage or volume loss at another site. We observed only one compartmental association (medial femoral BMLs predicted medial tibial cartilage defect increases). The site-specific nature of most associations suggests BMLs may be having an effect on the cartilage directly adjacent to the BML. BMLs may precede cartilage damage by altering cartilage nutrition resulting in cartilage defects. Furthermore, BMLs are made of a mix of cell infiltrates [28,29] and possible cross-talk between subchondral bone and cartilage [30] could induce catabolism of the cartilage. However, it is also possible that BMLs may be a secondary phenomenon as a result of cartilage damage. Indeed, this is the first study to demonstrate that baseline cartilage defects predicted site-specific BML progression. After further adjustment for meniscal damage this relationship was seen at the medial tibial and lateral femoral sites. Again we observed only one compartment association (lateral tibial defects predicted lateral femoral BML increases). Cartilage defects may exert an effect on the underlying bone by increased load transmission to the bone, resulting in BMLs. Alternatively, BMLs and cartilage defects may not necessarily drive one another, although it is possible. They may co-occur in the pathway towards increased disease. Therefore, it remains unclear whether BMLs precede, accompany, or follow cartilage damage and volume loss in OA [18].

Previous studies have shown that cartilage defects predict cartilage loss [31-33]. In this study, baseline cartilage defects predicted cartilage volume loss at the medial tibial site only; however, this did not persist after adjustment for meniscal damage. There was a trend towards cartilage defects predicting cartilage volume loss at the medial femoral site, independent of site-specific BMLs and meniscal damage. Baseline BMLs predicted cartilage volume loss at three of the four sites, independent of site-specific defects and meniscal damage. This demonstrates that BMLs were better than cartilage defects at predicting cartilage volume loss. Additionally, there was an interaction between baseline cartilage defects and BMLs on tibial cartilage volume loss at the medial and lateral sites, with a much greater rate of tibial cartilage volume loss when both larger defects and BMLs were present at the same site. This supports a previous study, which used finite element modeling to examine the effect of osteochondral defects on the knee joint [34]. They found that cartilage alterations were further exacerbated when bone damage was combined with base cartilage split and absence of vertical collagen fibrils [34].

Cartilage volume, cartilage defects, BMLs, and meniscal damage were all measured independently. This is a strength of the study. However, this study has potential limitations as well. First, follow-up MRI scans were only available on a subsample of the full TASOAC study. However, there were no significant differences between the subjects included in the current study and those in the rest of the cohort in regards to demographics, baseline cartilage defects, BMLs, and cartilage volume. Second, we used a study design with two time points to examine whether BMLs predicted cartilage defect progression and whether cartilage defects predicted BML progression. A study with more than two time points may give more insight into the causal pathways between BMLs and cartilage damage. Third, knee malalignment has been postulated as one factor explaining, at least in part, the association between BMLs and cartilage loss in OA [2,13]. However, in a previous study we found that baseline malalignment was not associated with subsequent loss of cartilage volume or progression of chondral defects [35]. Our current results suggest that malalignment may not be the driving factor, considering femoral BMLs did not predict tibial cartilage volume loss. If the effect of BMLs on cartilage volume loss was biomechanical, compartment-specific associations between BMLs and cartilage volume loss would be expected. However, because we did not have information about malalignment we cannot conclusively say whether or not malalignment plays a role in the associations we have seen. Fourth, cartilage defects were assessed on T1-weighted gradient-recalled echo (GRE) MR images and some research groups propose that GRE type sequences are less suited to detect cartilage defects [36]. We have recently published a letter to the editor of *Arthritis & Rheumatism *to address this issue [37]. There is evidence to demonstrate that GRE-type sequences are accurate and reliable for detecting cartilage defects with high sensitivity and specificity compared to arthroscopic results [38-40]. While our measure of cartilage defects may contain some measurement error and misclassification, it is likely to be random and would dilute the effects we see, thus reducing our ability to detect significant findings. Last, BMLs were read on T2-weighted images using a scoring system which is widely-published [3,41-43]; however, we have been made aware that scoring BMLs based on how many slices they appear on may bias towards flat but shallow lesions. For this reason, we extended our observation and performed a separate analysis in which BMLs were also scored by a different research group using a modified version of the WORMS method on T1-weighted images. Reading BMLs on T1-weighted MRI sequences may result in a more conservative analysis; however, d'Anjou *et al. *recently published a letter to the editor of *Osteoarthritis and Cartilage *to address whether non-cystic BMLs can be accurately measured using GRE type sequences [44]. The authors presented evidence to demonstrate that GRE type sequences are equally effective in detecting the presence of BMLs compared with T2-weighted fast spin echo sequences [44]. The results of the current study using both scoring systems with the two sequence types were highly consistent providing reassurance that our findings are valid.

## Conclusions

Baseline BMLs predicted site-specific defect progression and cartilage volume loss in a dose-response manner, which suggests BMLs may have a local effect on cartilage homeostasis. Baseline cartilage defects predicted site-specific BML progression, which may represent increased bone loading adjacent to defects. These results suggest BMLs and cartilage defects are interconnected and play key roles in knee cartilage volume loss; thus, both should be considered targets for intervention.

## Abbreviations

Β: beta-coefficent; BMI: body mass index; BMLs: bone marrow lesions; CI: confidence interval; CV: coefficient of variation; GEE: generalized estimating equations; GRE: gradient-recalled echo; ICC: intraclass correlation coefficient; MRI: magnetic resonance imaging; OA: osteoarthritis; OR: odds ratio; pa: per annum; TASOAC: Tasmanian Older Adult Cohort; WORMS: Whole-Organ Magnetic Resonance Imaging Score.

## Competing interests

Dawn Dore, Ashleigh Martens, Stephen Quinn, Changhai Ding, Tania Winzenberg, Guangju Zhai, Flavia Cicuttini, and Graeme Jones declare that they have no competing interests. Jean-Pierre Pelletier and Johanne Martel-Pelletier are consultants for and shareholders in ArthroVision Inc. François Abram is an employee of ArthroVision, Inc.

## Authors' contributions

DD and AM carried out analysis and interpretation of data, and prepared the manuscript. SQ participated in analysis and interpretation of the data, and critically revised the manuscript. CD designed and carried out the study planning, carried out data collection, participated in interpretation of data, and critically revised the manuscript. TW participated in interpretation of the data, and critically revised the manuscript. GZ and FA carried out data collection and critically revised the manuscript. JPP and JMP participated in the study planning, carried out data collection, and critically revised the manuscript. FC designed and carried out the study planning, participated in interpretation of data, and critically revised the manuscript. GJ designed and carried out the study planning, participated in analysis and interpretation of the analysis, and critically revised the manuscript. All authors have read and approved the final manuscript.
